# Phase 2b Study of an Ad26.RSV.preF Vaccine for Prevention of RSV-mediated Respiratory Tract Disease in Older Adults

**DOI:** 10.1093/geroni/igab046.3723

**Published:** 2021-12-17

**Authors:** Ann Falsey, Kristi Williams, Efi Gymnopoulou, Stephan Bart, John Ervin, Els De Paepe, Christy Comeaux, Esther Heijnen

**Affiliations:** 1 University of Rochester School of Medicine, Rochester, NY, USA, Rochester, New York, United States; 2 Janssen Research and Development, Spring House, PA, USA, Spring House, Pennsylvania, United States; 3 Janssen Infectious Diseases, Beerse, Belgium, Beerse, Antwerpen, Belgium; 4 Optimal Research, LLC/Synexus Clinical Research/AES, Woodstock, MD, USA, Rockville, Maryland, United States; 5 AMR Kansas City, Kansas City, MO, USA, Kansas City, Missouri, United States; 6 Janssen Vaccines & Prevention BV, Leiden, The Netherlands, Leiden, Zuid-Holland, Netherlands

## Abstract

Respiratory syncytial virus (RSV) may cause serious lower respiratory tract disease (LRTD) in older adults, and there is currently no licensed vaccine. CYPRESS (NCT03982199) is a randomized, double-blind, placebo-controlled Phase 2b proof-of-concept trial of an Ad26.RSV.preF-based vaccine for the prevention of RSV-mediated LRTD in older adults. Adults aged ≥65 years were randomized 1:1 before the RSV season to receive Ad26.RSV.preF-based vaccine or placebo. Acute respiratory infection symptoms were collected through a patient eDiary and/or clinician assessment until the end of the RSV season. The primary endpoint was the first occurrence of RTPCR-confirmed RSV-mediated LRTD according to any of 3 case definitions: (1) ≥3 symptoms of lower respiratory tract infection (LRTI), (2) ≥2 symptoms of LRTI, or (3) ≥2 symptoms of LRTI or ≥1 symptom of LRTI with ≥1 systemic symptom. Immunogenicity was assessed in a subset of approximately 200 participants. A total of 2891 participants in each study arm received study treatment. Vaccine efficacy was 80% (94.2% CI, 52.2-92.9%), 75% (50.1-88.5%), and 69.8% (43.7-84.7%) for case definition 1, 2, and 3, respectively (all P <0.001). In the vaccine arm, geometric mean fold increase in antibody titers 14 days after vaccination was 13.5 for RSV neutralizing antibodies and 8.6 for RSV prefusion F-specific binding antibodies, and median frequency of RSV-F-specific INFγ T-cells increased from 34 to 444 SFC/10^6 PBMC; no relevant changes were observed in the placebo arm. The Ad26.RSV.preF-based vaccine was highly effective against RSV-mediated LRTD through the first RSV season and elicited robust immune responses in older adults.

